# An Interesting Case of Alpha-Fetoprotein (AFP)-Producing Pancreaticoduodenal Tumor

**DOI:** 10.7759/cureus.59384

**Published:** 2024-04-30

**Authors:** Ani Gvajaia, Michael Imeh, Ali Raza

**Affiliations:** 1 Internal Medicine, New York City Health and Hospitals Corporation (NYCHHC) Lincoln Medical and Mental Health Center, Weill Cornell Medical College, Bronx, USA; 2 Surgical Oncology, New York City Health and Hospitals Corporation (NYCHHC) Lincoln Medical and Mental Health Center, Weill Cornell Medical College, Bronx, USA

**Keywords:** high afp-producing pancreaticoduodenal cancer, different mechanisms of afp-producing cancers, molecular mechanism of afp-producing adenocarcinomas, high afp in gastrointestinal tumor, pancreaticoduodenal cancer

## Abstract

Alpha-fetoprotein (AFP) is considered one of the best-known predictive serum markers, playing a crucial role in cancer investigation and subsequent treatment. In most adult cells, the production of this marker is suppressed after embryogenesis. However, its increased level raises concerns about underlying malignant conditions, which provide a valuable diagnostic tool for medical professionals in oncology. The existing AFP-producing adenocarcinomas exhibit unique clinical characteristics, including high malignancy and early metastatic potential, which result in poorer outcomes. To illustrate these characteristics, we decided to describe a case report of a 70-year-old African American female with a significantly elevated level of AFP. Further pathology results confirmed a duodenal adenocarcinoma versus adenocarcinoma from the pancreas. While AFP-producing adenocarcinoma has multiple underlying molecular mechanisms that correlate with poor prognosis, definitive treatment based on molecular pathways has yet to be defined. Therefore, further research is needed for new therapeutic modalities.

## Introduction

Alpha-fetoprotein (AFP) is a glycoprotein encoded in chromosome 4q25, which has multiple ligand binding sites and is generally synthesized under two clinical contexts. During pregnancy, it is synthesized in the yolk sac, fetal liver, and gastrointestinal tract and involved in nutrient transport and ontogenic processes. Additionally, AFP can be synthesized in different adult tumors of mixed mesodermal/endodermal origin [1,2]. Clinically, AFP is considered one of the best-known predictive serum markers and is an essential and common target in cancer investigation and subsequent treatment [3].

Embryologically, AFP receptors (AFPR) on fetal cells internalize the AFP ligand complex via AFPR endocytosis, a function regained in cancer cells. In contrast to the embryogenic/fetal stage, the AFPR is absent in normal adult cells, with the exception of a small population of regulatory immune cells: myeloid-derived suppressor cells (MDSC). These unique features make AFP a fascinating and multifunctional entity, confirming its involvement in immune regulation, cell growth, and differentiation. MDSC provides immunosuppressive function through the induction, proliferation, and recruitment of regulatory T cells, especially when they accumulate in the tumor [4].

Given that AFP is typically suppressed in most normal adult cells, the elevation of AFP levels in adult patients should raise significant concerns. This increase often indicates the presence of pathological conditions such as hepatocellular carcinoma, germ cell tumors, and rare cases of adenocarcinomas [5]. Notably, clinical investigations of AFP-producing adenocarcinomas have revealed a worrying trend toward high-malignant and metastatic potential, leading to poorer outcomes for patients with these tumors [5,6].

## Case presentation

Our case involves a 70-year-old African American female with a past medical history of hypertension and hyperlipidemia without any previous history of pancreatitis or alcohol use who initially presented into the emergency room with a 3-4-week history of epigastric pain associated with intermittent nausea, vomiting, and food intolerance. She noticed an increasing loss of appetite and unintentional weight loss of 23 lbs. over the last four months. She denied acholic stools, yellowing of her eyes or palms, or back pain.

Physical examination revealed a well-nourished, obese female without icterus or jaundice. Inspection of her abdomen showed no abnormality and demonstrated no distention, tenderness, or palpable masses. Her initial laboratory findings were significant for leukocytosis, anemia, pancreatitis, and a biliary obstruction with cholestatic hepatitis (Table 1).

**Table 1:** Laboratory findings. Initial lab findings with tumor markers. WBC: white blood cell; HGB: hemoglobin; ALP: alkaline phosphatase; AST: aspartate aminotransferase; ALT: alanine aminotransferase; AFP: alpha-fetoprotein; CEA: carcinoembryonic antigen; CA19-9: carbohydrate antigen 19-9

Lab findings	Value	Reference range
WBC	15.5↑	4.80-10.80×10^3^/mcl
HGB	9.6↓	12.0-16.0 g/dl
Lipase	1308↑	13-60 U/L
Total bilirubin	3.8↓	0.2-1.2 mg/dl
Direct bilirubin	2.5↑	0.00-0.30 mg/dl
ALP	217↑	35-105 U/L
AST	97↑	≤32 U/L
ALT	114↑	≤33 U/L
AFP	13,516↑	≤8.3 ng/ml
CEA	28↑	Non-smoker <3.9 ng/ml; smoker <5.5 ng/ml
CA19-9	100↑	≤35 U/mL

**Figure 1:**
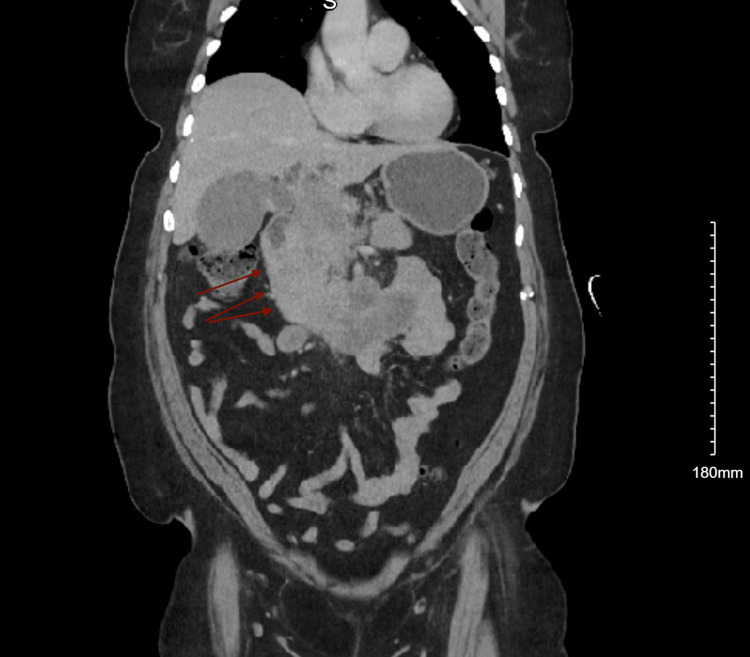
CT of the abdomen. Red arrows: non-discernible mass-like irregularity in the region of the pancreatic head.

**Figure 2:**
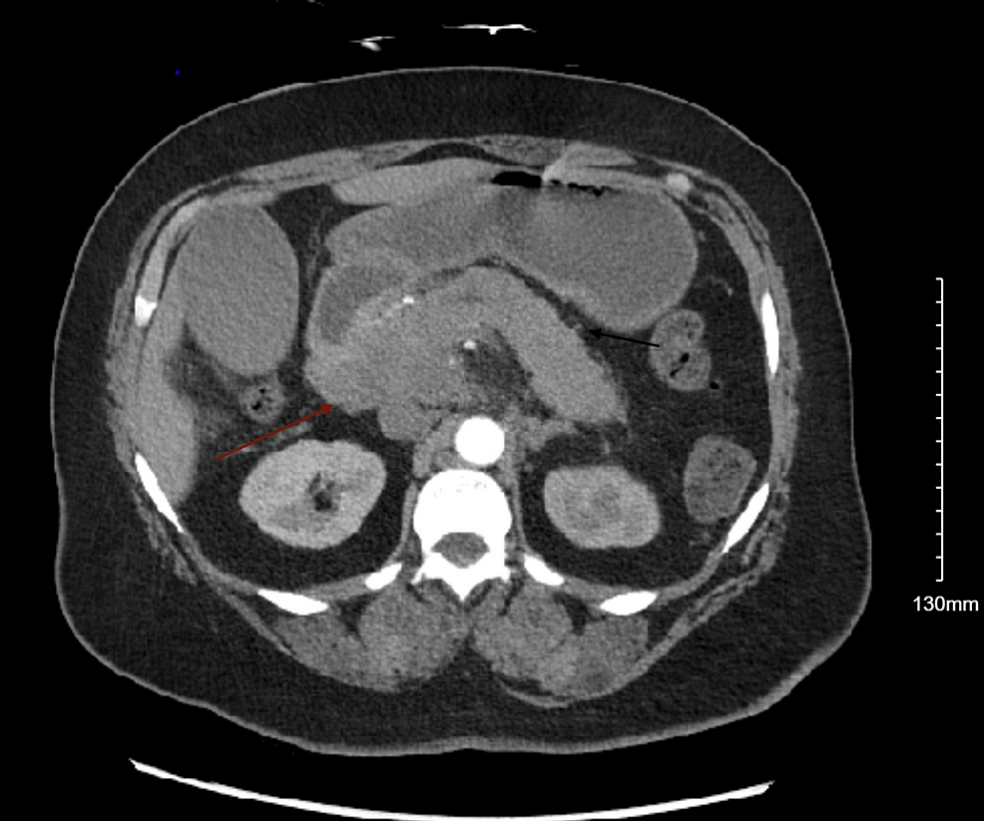
CT of the abdomen. Red arrow: pancreas head. There are diffuse thickening of the pancreas and significant surrounding fat stranding (black arrow), especially in the region of the pancreatic body, which is concerning for pancreatitis.

Initial imaging by way of CT of the abdomen/pelvis showed a mass-like irregularity with cystic changes in the region of the pancreatic head/body that measured 10.5 cm. A mildly dilated common bile duct and main pancreatic duct were also present, which increased the concern of underlying pancreatic neoplasm (Figure 1 and Figure 2). This prompted serology that was remarkable for elevations in AFP and compared to other markers, including a carcinoembryonic antigen (CEA)/carbohydrate antigen 19-9 (CA19-9) (Table 1).

**Figure 3:**
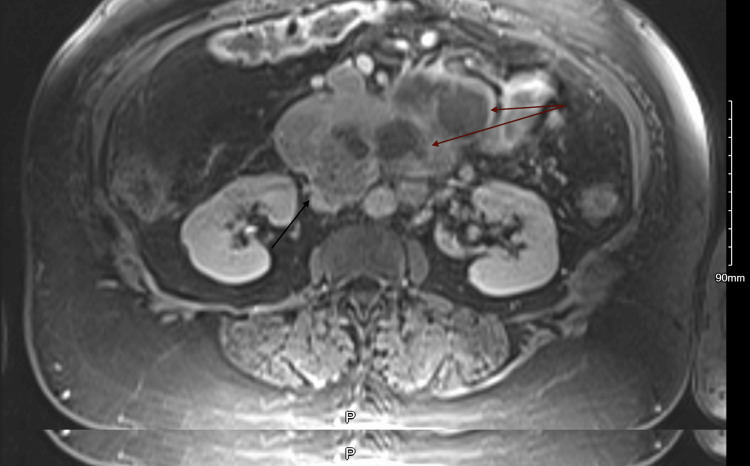
MRI of the abdomen. Black arrow: bulky pancreatic head without a discrete mass. However, the pancreatic head is contiguous with the duodenum without a clear fat plane concerning infiltrating disease. Red long arrows: along the uncinate process of the pancreas, there are ill-defined multiloculated cystic lesions.

After inpatient admission, gastroenterology and surgical oncology were consulted, and an endoscopic ultrasound (EUS) with endoscopic retrograde cholangiopancreatography (ERCP) was undertaken to assess for a presumed pancreatic cancer. A subsequent MRI of the abdomen was also performed and demonstrated a large pancreatic uncinate/head mass with the common hepatic artery (CHA) and right hepatic artery encasement, extension into the duodenal lumen, and portal vein (PV) and superior mesenteric vein (SMV) occlusion (Figure 3). Additionally, multiple enlarged mesenteric and peripancreatic lymph nodes were noted.

**Figure 4:**
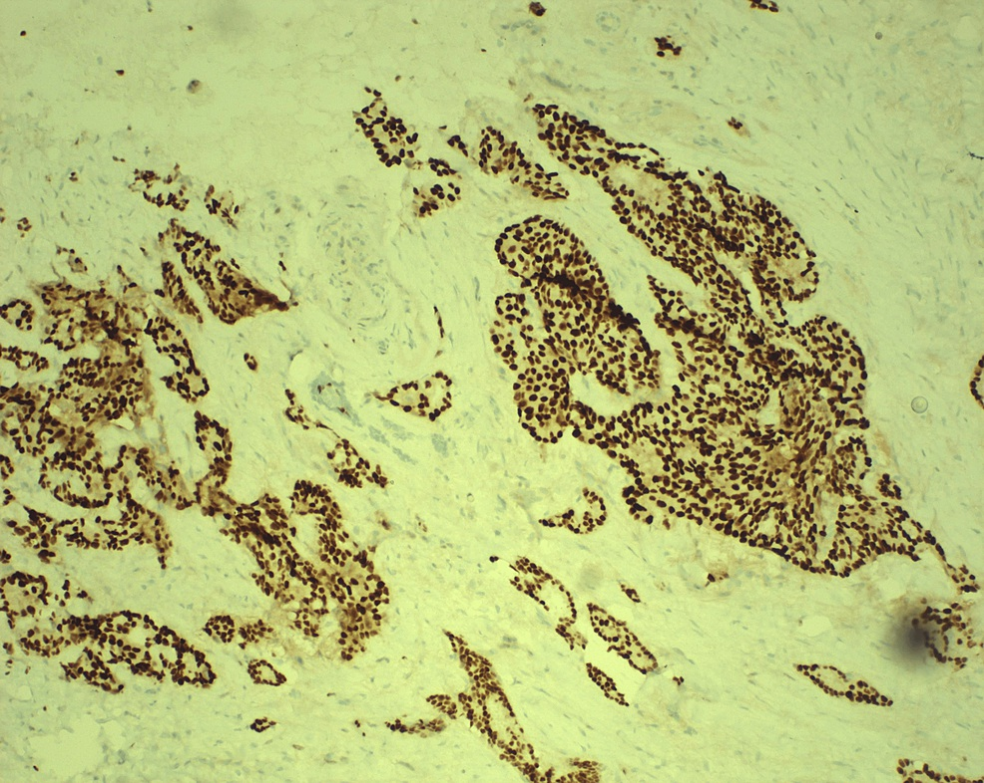
Slide 1. CDX-2 immunohistochemical stain is strongly positive for adenocarcinomas of intestinal origin, consistent with the rendered diagnosis.

**Figure 5:**
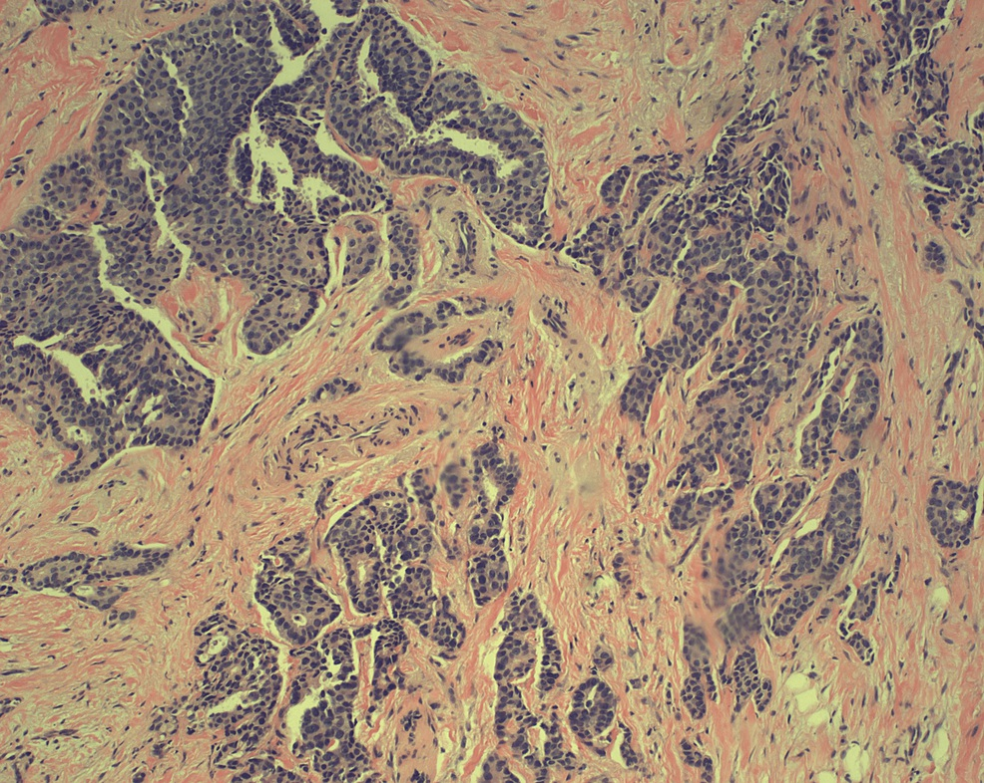
Slide 2. The section shows adenocarcinoma, moderately to poorly differentiated, consistent with either primary duodenal carcinoma or pancreatic carcinoma extending to the duodenum (hematoxylin-eosin, original magnification ×100).

An EUS was performed and demonstrated a presumed pancreatic tumor with a partial duodenal outlet obstruction. Imaging shows a 6x5 cm head of pancreas ulcerated mass starting at the duodenal angular region and extending into the second portion with 90% lumen occlusion. Additionally, the duodenal imaging with EUS showed an irregular mural thickening up to 2 cm, consistent with perilesional and celiac adenopathy. ERCP with stenting was attempted, and a large fungating duodenal mass with obliteration of the ampulla was demonstrated, precluding canalization and biliary stent placement. Biopsies were taken of the ulcerated region, and initial pathology returned with adenocarcinoma with moderate to poor differentiation (Figure 4 and Figure 5).

Subsequent tumor board discussion with a review of imaging demonstrated a locally advanced and unresectable tumor originating in the pancreatic head and second portion of the duodenum. The final pathology was reviewed, and the etiology of the tumor was difficult to discern, representing either a primary duodenal carcinoma or an adenocarcinoma of the pancreas. Systemic and definitive chemotherapy was initially chosen with gemcitabine and Abraxane and was later revised to single-agent gemcitabine with a 20% dose reduction, given the mild biliary obstruction. The patient, during her inpatient course, had intermittent episodes of nausea and food intolerance. The surgery went on to perform a gastrojejunostomy for palliative intent, given the concern for a complete future duodenal obstruction. After that, the patient tolerated a diet and was discharged with a plan for outpatient systemic therapy.

## Discussion

AFP-producing adenocarcinoma is a non-hepatocellular adenocarcinoma that secretes the AFP glycoprotein. Several clinical characteristics are associated with AFP-producing adenocarcinomas and include early multi-organ metastasis, especially liver/lymph node metastases at the time of diagnosis, as well as multidrug resistance to therapy. Both of these lead to a rapidly worsening clinical state after diagnosis and correlate to a much inferior prognosis in AFP-producing adenocarcinoma originating from the gastrointestinal tract (APA-GI) compared to non-AFP-producing adenocarcinomas of the gastrointestinal tract [7]. Therefore, AFP positivity is a significant negative predictor of overall survival. 

AFP-producing adenocarcinomas consist of two histological subtypes, hepatoid adenocarcinoma and adenocarcinoma, with fetal gut-like features, and both of these subtypes, in some cases, can co-exist. The number of case reports of AFP-producing adenocarcinomas in various organs is increasing, highlighting the need for updated knowledge in this area. However, the immunohistochemical characteristics and molecular diagnostics have been studied; the molecular mechanisms underlying the development of AFP-producing adenocarcinomas remain unclear [6]. One of the presumed genetic mechanisms of the development of AFP-producing adenocarcinomas is based on the transcription factor forkhead box A (FoxA), also known as hepatocyte nuclear factor (HNF)-3. There are three different isoforms coded in the other genes, i.e., FoxA1 (HNF-3α), FoxA2 (HNF-3β), and FoxA3 (HNF-3γ). FoxA1 and FoxA2 are involved directly in the organogenesis of the endodermal derivatives and ensure the development of anterior definitive endodermal tissue (lung, thyroid, and thymus progenitors) and posterior definitive endodermal tissue development (liver, pancreas, and intestinal progenitors) [6,8].

There are two main theories regarding AFP-producing cancers. One could consider cancer cells acquiring the ability to produce AFP during cancer cell differentiation, invasion, and proliferation. The theory was confirmed by 19 AFP-producing gastric adenocarcinomas (AFPGA) gene analyses where multiple foci were microdissected and loss of heterozygosity (LOH) analysis was performed. Based on the study's conclusion, 13q LOH was often associated with AFP neoplastic foci. Knowing the fact that the same 13q LOH is commonly deleted in AFPGA and is also frequently deleted in AFP-producing hepatocellular carcinoma (AFPHCC) strengthens the possibility that high fractional allelic loss in 13q could be responsible for the regulation of AFP synthesis; therefore, 13 qLOH and additional alteration of the remaining allele may silence or downregulate the gene and produce AFP [5,9,10].

Otherwise, signaling pathways mediated by hepatocyte growth factor (HGF)/c-Met could be responsible for AFP production. This underscores the role of HGF/c-Met signaling pathways in the development of AFP-producing adenocarcinomas, providing a clear link between these pathways and the production of AFP. Several studies confirmed that the integrity of HGF and its receptor (c-Met) could regulate cell proliferation and motility, promoting tumor progression. One clinical study by Amemiya et al. found that the c-Met expression level is higher in patients with AFP-producing gastric cancers compared to stage-matched gastric cancers that did not produce AFP [11]. Additionally, a case study revealed all three cases of AFP-producing colorectal cancer in which activation of the HGF/c-Met/the transcription factor c-Myc signaling pathway was observed. This may support the conclusion that autocrine HGF/c-Met activation may induce the dedifferentiation of standard adenocarcinoma cells into a stem cell state to produce AFP or hepatoid differentiation [12,13].

Dysregulation of the paracrine HGF/c-Met activation could also provide oncogenesis and tumor progression in several cancers and promote aggressive cellular invasiveness associated with tumor metastasis. HGF/c-Met activation may induce the dedifferentiation of common adenocarcinoma cells, which revert to a stem cancer cell phenotype and produce AFP or hepatoid differentiation. In adenocarcinomas, high activity of the Wnt/β-catenin pathway is observed. Myofibroblast-secreted HGF can activate β-catenin-dependent transcription and colon cancer stem cell clonogenicity. 

Moreover, myofibroblast-secreted HGF can restore differentiated tumor cells from the cancer stem cells [14,15].

Additional features, such as a high expression rate of vascular endothelial growth factor (VEGF) and its isoform VEGF-C, were noticed in AFPGA when immunohistochemical analysis was done in 26 patients with AFPGA compared to patients with gastric adenocarcinomas without AFP production. The expression rate of VEGF-C was significantly higher in the AFP-positive group than in the AFP-negative group (P<0.01). Therefore, the angiogenic and lymphangiogenic function of VEGF and its isoform VEGF-C could be associated with tumor progression [16].

Other various molecular targets have been correlated with AFP expression as well. An oncofetal SALL4-zinc finger DNA-binding protein maintains self-renewal and drives reprograming somatic cells toward pluripotent embryonic stem cells. AFP is closely associated with SALL4 expression in gastric carcinoma (both, P<0.0001) [17]. 

Lastly, rapid tumor proliferation, with highly expressed progression marker Ki67 with low apoptotic index and high microvessel density (neovascularization), was noticed in the AFPGA compared to the AFP-negative gastric cancers (P<0.01) [18].

In relation to pancreatic cancers, AFP-producing pancreatic cancer is usually derived from acinar cells; AFP-producing adenocarcinoma from ductal cells is relatively rare. One case report documented elevated tumor markers of AFP and PIVKA (34,748 ng/ml and 474 mAU/ml, respectively, with normal level CEA and CA19-9) in a patient with multiple liver nodules and a mass in the head of the pancreas with unclear origin. Diagnosis of pancreatic ductal carcinoma (stage IVb) by liver biopsy was ultimately obtained [19].

Overall, 21 AFP-producing duodenal carcinomas have been documented. Recently, an autopsy case about an AFP-producing large duodenal adenocarcinoma was published with a significantly high level of AFP (42,078.4 ng/ml). In contrast, CEA and CA19-9 levels were normal. Pathological results confirmed a diagnosis of AFP-producing poorly differentiated adenocarcinoma [20].

## Conclusions

In our case, pathology results demonstrated a duodenal adenocarcinoma versus adenocarcinoma from the pancreas with a significantly elevated AFP of 13,516 ng/ml. Clinical characteristics such as early metastatic disease were also reported in our case, given the large size of the tumor, its locally advanced state with PV thrombosis/hepatic artery encasement, and the presence of peripancreatic and mesenteric adenopathy. While multiple underlying molecular mechanisms of AFP-producing adenocarcinoma exist and correlate with poor prognosis, definitive treatment based on molecular pathways has yet to be defined, and further research for new therapeutic modalities is needed.
